# Trachoma: an underdiagnosed disease revealed by a survey carried out at Jaú, São Paulo

**DOI:** 10.1186/s12886-024-03302-2

**Published:** 2024-01-29

**Authors:** Luisa Fioravanti Schaal, Roberta Lilian Fernandes de Sousa Meneghim, Lucieni Cristina Barbarini Ferraz, Carlos Roberto Padovani, Cassiano Victoria, Silvana Artioli Schellini

**Affiliations:** 1 Surgical Specialties and Anesthesiology Department, Botucatu Medical School, State University of São Paulo– UNESP, Avenida Professor Mário Rubens Montenegro, Botucatu, São Paulo, 18618-970 Brazil; 2 Bauru State Hospital, Av. Eng. Luís Edmundo Carrijo Coube, 1-100 - Nucleo Res. Pres. Geisel, Bauru, SP 17033-360 Brazil; 3 Department of Biostatistics, Plant Biology, Parasitology and Zoology, Botucatu Institute of Biosciences – State University of São Paulo- UNESP, Rua Professor Doutor Antonio Celso Wagner Zanin, 250, Botucatu-São Paulo, 18618-689 Brazil; 4 Department of Animal Production and Preventive Veterinary Medicine, Faculty of Veterinary Medicine and Zootechnics of Botucatu, State University of São Paulo- UNESP, Rua Prof. Doutor Walter Maurício Correa S/N, São Paulo, CEP: 18618-681 Brazil

**Keywords:** Trachoma, Prevalence, Epidemiology surveys, Brazil

## Abstract

**Background:**

Trachoma is a notifiable disease in the state of São Paulo– Brazil. However, in Jaú, a municipality located in this state, in the last 10 years there are no records of cases. This study purpose is to assess if there are cases of inflammatory trachoma in schoolchildren aged 1 to 9 years in the municipality of Jaú as well if it is possible to detect clusters areas of the disease to establish elimination programs.

**Methods:**

An epidemiological study was performed in 2018, involving a stratified random sample of schoolchildren aged 1- to 9-year-old, from public day care centers and elementary schools in the municipality of Jaú. A trachoma screening following the criteria of the World Health Organization (WHO) and the distribution of cases was assessed using geoprocessing.

**Results:**

Four thousand-six hundred-nineteen children from 44 elementary schools were examined, and 126 children with active trachoma were detected, with an adjusted prevalence rate of 2.65%. The prevalence was higher (3.01%) in children aged 6- to 9-year-old compared to children aged 1-to 5-year-old (2.42%). There were clusters with a higher concentration of positive cases of the disease in three schools located in the neighborhoods with lower socioeconomic conditions.

**Conclusion:**

Inflammatory trachoma still present in children aged 1- to 9-year-old in the city of Jaú. The positive cases were located mainly in areas with low socioeconomic conditions. Health promotion with active search and periodical treatment must be planned to fight this important blinding cause, that persists in our municipality.

## Background

Trachoma is considered the number one infectious cause of blindness. Children are the reservoir of infection, while blindness, occurs after repeated episodes of infection [1]. From 2002 to 2007 the disease prevalence in the southeast region of Brazil was 4.4%, and 4.1% in the state of São Paulo [2]. In 2016, a Brazilian survey including 27 states showed a prevalence of inflammatory trachoma in schoolchildren of 5%, ranging from 1.5 to 9.0% according to the geographic region [3].

The disease was assumed as eliminated in São Paulo state, remaining without surveillance. However, in the year 1986 a screening performed in the Northeast region of the state revealed a prevalence of 7.2% [4]. Other surveys carried out in other cities of the state, revealed prevalence from 9.6% in Guaraci (1989) [5] and 1.5% in Franco da Rocha (1989) [6].

The prevalence of inflammatory trachoma in schoolchildren in the city of Bauru, located in the central region of the state, was 6.5% in the year 1936, remaining with no record of the disease from 1984 to 1990 [4]. However, a survey in the year 1991 showed 19 cases of trachoma in children aged 1 to 10 years old [4] and a prevalence of 3.8% was detected in 2006 in schoolchildren from 6 to 14 years old [7]. Botucatu is other city located in the same region, with a prevalence of 11.9% of active cases in children from 4 to 11 years old reported in 1992 [8] and 3.14% in children aged 6 to 10 years old in 2013 [9].

Jaú is located in the same area of Bauru and Botucatu. However, despite being a disease of compulsory notification in the state of São Paulo [10], no cases of trachoma were reported in the past 10 years. With the region historical backgrounds, the question is if trachoma is controlled in the city or if it is underdiagnosed. An active search should be used to answer this question.

So, the aim of the present study was to search for inflammatory trachoma in schoolchildren from 1 to 9-year- old in the city of Jaú, to establish the prevalence of cases as well as to identify possible trachoma clusters areas of the disease in the municipality.

## Methods

An epidemiological study, searching for the prevalence of trachoma in schoolchildren from the city of Jaú, São Paulo - Brazil, and geoprocessing of the positive cases was performed. The Botucatu Medical School Ethics Committee for Human Research (ID 2.421.676) approved this study protocol and parents, or legal tutor signed the informed consent for participation.

### Sample calculation

the sample involved schoolchildren from 1- to 9-year-old enrolled in public daycare centers or elementary schools during the year of 2018 in the city of Jaú, São Paulo. The city has 57 municipal schools, 14 state schools and 32 private schools [11]. According to the 2010 national census [12], in the year 2018 there were approximately 16,800 children from 0- to 9-year-old living in Jaú, 14,057 children enrolled in public schools, 2,743 in private school or did not attend school yet, divided in 17 elementary schools (children from 6 to 9 years) and 40 daycare centers (children from 1to 5 years), being 8,298 children ageing between 1 and 9 years old, and 5,759 ageing 10 years or older.

The sample size was calculated based on inflammatory trachoma prevalence detected in other cities from the same region [7, 9], taking an estimated prevalence of 3%, with confidence interval of 95% and estimation error of 20%, adding 5% to minimize losses, the minimum of 4,795 children should be examined, being 1,932 from daycare centers and 2,863 from elementary schools, respecting the region proportion. Forty-four schools were selected, being 27 daycare centers and all the 17 elementary schools, with a total of 7,853 enrolled students.

**Fig. 1:**
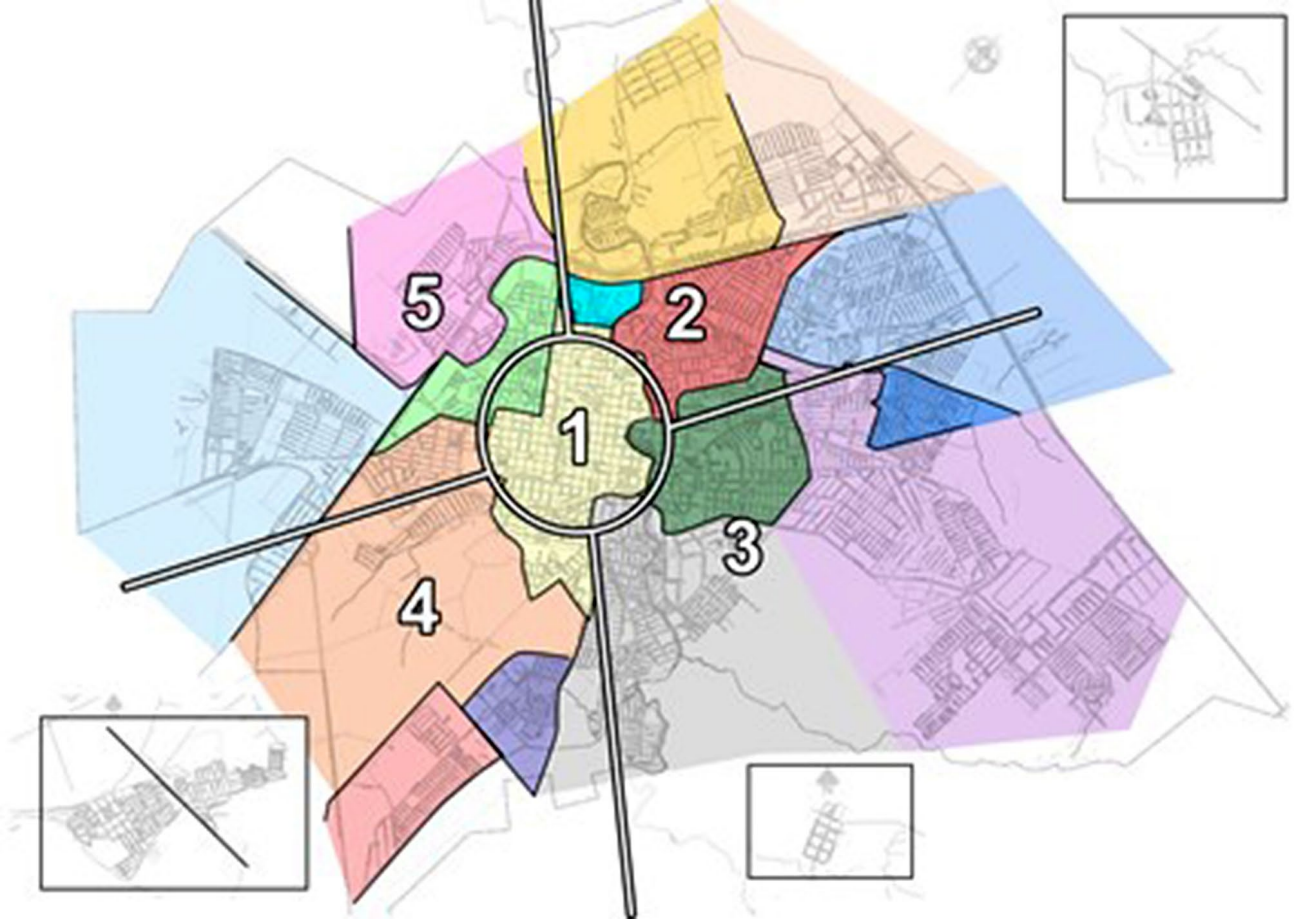
Map of the five studied regions with the schools’ coverage, Jaú − 2018

For this study, the city of Jaú was divided in one central region and four peripheral regions (Fig. 1) based on school’s coverage map from the Education Department of Jaú. All sectors were represented equally within the sample and all schools were considered to participate. The daycare centers and elementary schools for each region were numbered and randomly selected. After the school visit, if the sample size was not reached another school was selected randomly until the needed sample size for that region and age group was achieved. All children aged from 1- to 9-year-old from each selected school were considered for the evaluation.

### Study location description

Jaú is a Brazilian municipality located in the central-west region of *São Paulo state*, at 541 m of altitude and 296 km far from the capital of the state. It is formed by the city, including rural districts of *Vila Ribeiro, Potunduva* and *Pouso Alegre de Baixo*. Its population was estimated of 151,881 inhabitants by the IBGE (Instituto Brasileiro de Geografia e Estatística) in the year of 2020. The city is an important center of industrial and agriculture development, standing out for the number of women shoe factories, with a Gross domestic product (GDP) per capita in 2018 of US$ 6,371.07. The percentage of children enrolled in the schools in the municipality is 97.8% and 98.4% of houses have water and sewage coverage. The city´s HDI (Human Development Index) is 0.778, with 28.39% of its population in low social vulnerability and 9.83% in high social vulnerability [13, 14].

### Eye exam method

the evaluation was performed in the schools, during the period of classes, between October and November of 2018, by three trained examiners for the diagnosis of trachoma, following the guidelines of the World Health Organization (WHO) [15, 16]. Clinical exam was performed by four trained ophthalmologists, using 2.5 times magnification loupes and a flashlight. The examination searched for changes in the eyelids, eyelashes, conjunctiva (tarsal and bulbar) and cornea. The upper eyelid was manually everted, and the superior tarsal conjunctiva was carefully examined.

Trachoma was diagnosed according to the WHO criteria for epidemiological detection of the disease: trachoma follicular was characterized by presence of five or more follicles measuring more than 0.5 mm in the center of the upper eyelid tarsal conjunctiva; trachoma intense was characterized by thickening of the conjunctiva shadowing half of the deep tarsal vessels [17, 18].

A child was considered a case when clinical diagnose met the cited criteria. All the detected cases were notified to the Epidemiological Surveillance of Jaú. Parents, or tutors, and the other residents of the same household, were gathered to receive information about the disease, to be examined and treated for the disease according to the WHO guidelines, with a single dose of oral 1 g Azithromycin for children weighing more than 40Kg or 20 mg/Kg of the oral suspension for the ones weighing less than 40Kg [19]. Treatment was provided free of charge by the municipality epidemiological surveillance department. The reunion was taken place in the evening to encourage the presence of all and posteriorly an active search for those missing treatment was performed. After six months of the treatment, all cases were reexamined for a treatment control.

### Statistical analysis

the data were transferred to an Excel spreadsheet and submitted to statistical analysis. Prevalence was calculated according to the school region in the municipality and descriptive analysis was performed using the Goodman test for contrast between binomial proportions with confidence interval of 95% [20]. Descriptive statistics was also calculated according to the region, age and rural or urban area [21]. All conclusions were discussed with a 5% significance.

### Geoprocessing

the distribution of the cases in the municipality was studied using the home address of the affected children and the Water and Sewage Department data to assess if there were clusters areas of the disease. Geoprocessing was performed using the Bucuresti QGIS 3.12.3 Software. For data import the Planimetric Datum SIRGAS 2000/UTM zone 22 S was used. For point density analysis the quartic Kernel interpolator was used, with the QGIS of the HEATMAP algorithm, considering a fixed radius of 1000 m.

## Results

A total of 4,619 children were examined, corresponding to 96.3% of the required sample and 58.8% of the total of children enrolled in the selected schools. The distribution of the participants according to age is presented in Table 1, being 1,682 children between 1- to 5-year-old and 2,937 between 6- to 9-year-old corresponding to 87% and 102.5% of the intended sample, respectively.

One-hundred-twenty-six cases of active trachoma were detected, being 119 follicular (94.5%), and seven trachoma intense (5.5%), corresponding to an adjusted prevalence of 2.65% of inflammatory trachoma in Jaú. Seventeen cases were living in the rural area (13.49%), with no statistical difference in trachoma prevalence between urban and rural areas (*p* > 0,05). There was also no statistical difference between the regions of the city (Table 1).

**Table 1:** Distribution of participants according to age, region and prevalence descriptive measures in Jaú, 2018

	Region of the city	1	2	3	4	5	Total
Examined sample (percentage of required sample)	Elementary school (6 to 9 years-old)	217 (123.3%)	1135 (108%)	582 (100.9%)	337 82%)	666 (102.8%)	2937 (102.5%)
	Daycare centers (1 to 5 years-old)	115 (97.4%)	662 (93.4%)	312 (80.2%)	285 (102.5%)	308 (70.5%)	1682 (87%)
Adjusted Prevalence (%)	Median	4.17	2.48	2.90	3.50	1.61	2.66
	Average	2.93	2.27	3.04	3.38	2.26	2.65
	Standard deviation	2.54	2.48	1.55	3.17	2.84	2.35

*Numbers represent the region of the city showed in the map in Methods

The distribution of cases by age group showed that 35 cases were detected in children with 1- to 5-year-old, and 91 cases in children with 6- to 9-year-old, revealing a respective prevalence of 2.42% and 3.01%, with statistic significant difference (*p* < 0.05%).

The concentration of positive cases was distributed in three elementary schools: EMEF Vereador Angelo Ronchesel, with 10 cases, EMEF Isa Rosa Meireles Name with 12 cases and EMEF Pádua Sales, with 10 cases.

**Fig. 2:**
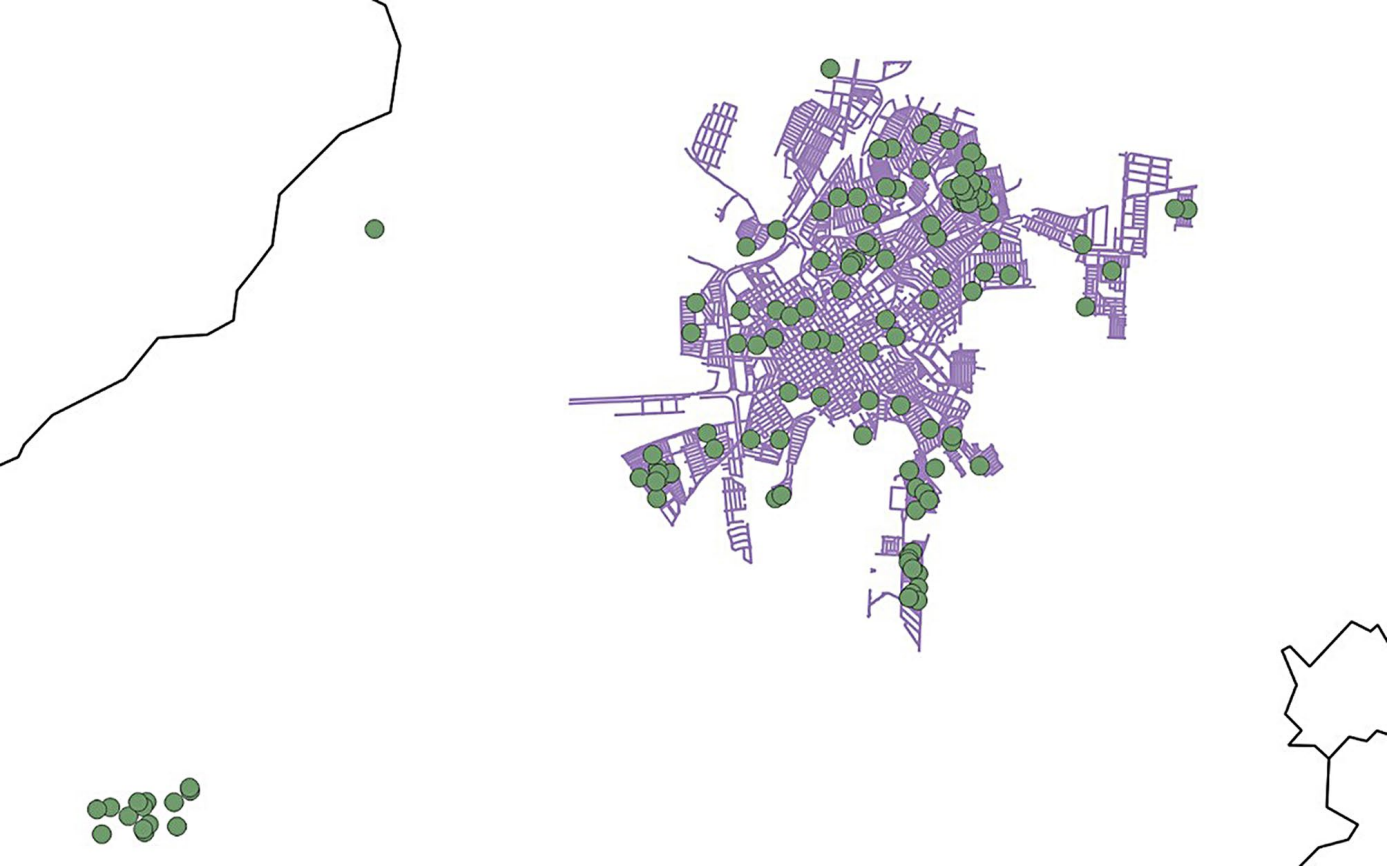
– Distribution of children with trachoma in Jaú municipality, 2018. Green dots representing each case

The address of the affected children was considered to estimate the distribution of cases the disease in the municipality (Fig. 2) as follow: Jardim Pedro Ometto, Jardim Orlando Ometto, Jardim Cila de Lúcio Bauab, Jardim Padre Augusto Sani and Potunduva district with 17, 16, 10 and 13 cases respectively.

**Fig. 3:**
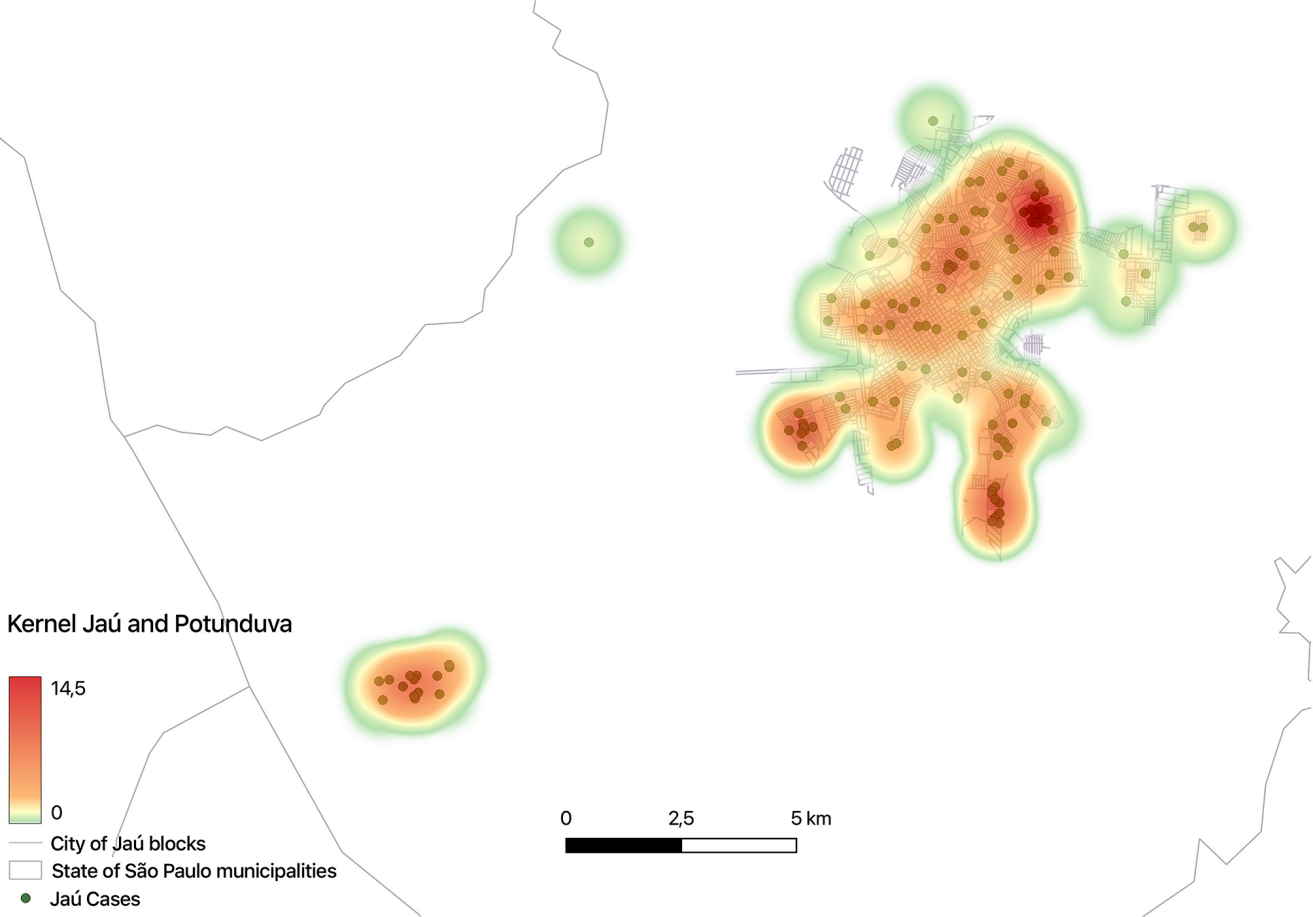
Distribution of the positive cases of trachoma in a concentration map in Jaú municipality − 2018

Geoprocessing of affected children in their respective place of residence demonstrated areas of disease concentration, highlighted by hot colors in the colored map (area of higher concentration are registered in red) (Fig. 3).

Even though the importance of the reunion with parents/tutors for explanation about the disease and treatment was reinforced, only 74 (58.7%) cases and their respective parents/tutors attended to receive the oral medication. One reason for no adherence detected in 4% of the children was they had already moved to another city.

## Discussion

The total prevalence of active trachoma in schoolchildren from 1- to 9- year-old in the city of Jaú was 2.65%, similar to the neighbor cities of Bauru and Botucatu [7, 9].

The sample size for children populational studies in endemic trachoma areas is calculated considering estimated prevalence of 10% of inflammatory trachoma [22]. However, we used the prevalence of 3% based on the previous studies performed in two cities from the same region [7, 9].

Less expensive and quickly trachoma surveys are the ones approaching children in the period of school permanency as we performed, since children are all in the same space. The high number of schooling children as observed in Jaú municipality is in favor of this method. However, a door-to-door search even though more expensive and time consuming, is the best one to detect positive trachoma cases.

Though 96.3% of the intended sample was examined, the percentage of examined sample from 1- to 5- year-old was slight lower (87%) probably because in this age, children´s attendance to daycare centers is optional for parents working outside and at this age group, children are not cooperative to ocular exams, absentees’ rate is higher, contributing more for not reaching the required sample.

The low prevalence of inflammatory trachoma in Jaú probably is a result of the good socioeconomic indicators in this municipality, similar to observed in the developed countries, with solid infrastructure, good HDI, with vast water and sewage coverage, and located in the most privileged economically state of Brazil. These factors influence the prevalence rate, with regions with lower HDI having grater prevalence of trachoma in children [3].

A significant difference was observed in the prevalence rate, being the prevalence higher in children age group from 6 to 9- years- old (3.09%) than 1 to 5-years- old (2.08%). Although our study detected higher rates of trachoma in older children, the national Brazilian inquiry showed smaller prevalence in older children [23]. 

Compared to the neighboring cities if Bauru and Botucatu, the disease index in the region is probably in a decrease [7, 9]. Our prevalence was also inferior to another national survey performed in Brazil, which detected an 8.3% rate [23]. Studies carried out in other Brazilian states pointed prevalence of 12.5% in children from 1 to 9 years in Brasilia [24], 4.5% in Roraima [25], and 4.7% in children from 7 to 15 years in Minas Gerais state [26]. A screening study for the disease in the city of São Paulo showed a 4.7% in the year 2006 [27]. However, in indigenous population, the prevalence may reach to 34.5% [28].

There are studies reporting more common cases of trachoma living in the rural areas [26]. However, after the rural exodus occurred in Brazil in the 50’s and 60’s, the rural population is small in the state of São Paulo and most of the children are living in urban areas [29]. In our sample 13.49% were considered rural residents but the prevalence was not higher in children living in rural area except in a rural district (Potunduva) where there was a great number of cases. Therefore, the risk factors associated to the disease in this place have to be identified.

The areas of disease concentration in Jaú were detected using geoprocessing, enabling a search for associated risk factors. The classic risk factors as water supply and sewage coverage are less relevant in Jaú because the good indicators in the municipality. The number of people living in the same household was not analyzed in our study, but Jaú does not differ from other regions of São Paulo state, with one or two children per family, much different from Nigeria were the average number of children in each household is greater than four, with the risk of trachoma 2.4 times higher there [30].

The school coexistence might play a role in trachoma transmission, and a concentration of cases was detected in a few schools. With geoprocessing, it was possible to detect areas of cases concentration nearby the schools with greater number of cases, confirming that the distribution of the disease is not equal even within the same municipality [27]. Therefore, the school agglomeration might have influenced in these areas of disease concentration, maintaining the presence of trachoma in Jaú. In addition, schools with a greater number of cases are in areas with lower socioeconomic conditions.

The suboptimal adherence for the treatment which was only 58.7%, even with all the facilities for parents and free treatment, was a weakness in our study. The low adherence to the study can result in cases not being detected, reducing the estimated prevalence. Undetected trachoma cases have a negative impact on the fight against this highly transmissible disease, but this fact is not exclusive to the present study, being reported in other trachoma prevalence research [31, 32].

Also, our study did not involve private schools, and this may have influence in our analysis.

The strength of the present study was the randomized sampling, the screening performed by three trained examinersʹ ophthalmologists for trachoma detection and the search being carried out following all WHO criteria, in a short period of time.

## Conclusion

The present study revealed inflammatory trachoma still present in Jaú, even with a low prevalence of 2.65%. The disease in Jaú municipality is under control but underreported, possibly because of lack of training and education of health professionals. Continuous training policy, education on the disease, active search, and treatment, are in need to prevent the increase of prevalence rate, even in a city with good HDI and conditions. Possible risk factors associated with the concentration of cases should be identified. Health promotion with active search and periodical treatment must be planned to fight this important blinding cause, that persists in our municipality.

## Data Availability

The datasets used and/or analyzed during the current study are available from the corresponding author on reasonable request.
